# Omeprazole Prevents Colistin-Induced Nephrotoxicity in Rats: Emphasis on Oxidative Stress, Inflammation, Apoptosis and Colistin Accumulation in Kidneys

**DOI:** 10.3390/ph15070782

**Published:** 2022-06-23

**Authors:** Mohammed Z. Nasrullah, Khalid Eljaaly, Thikryat Neamatallah, Usama A. Fahmy, Abdulmohsin J. Alamoudi, Hussain T. Bakhsh, Ashraf B. Abdel-Naim

**Affiliations:** 1 Department of Pharmacology and Toxicology, Faculty of Pharmacy, King Abdulaziz University, Jeddah 21589, Saudi Arabia; taneamatallah@kau.edu.sa (T.N.); ajmalamoudi@kau.edu.sa (A.J.A.); abnaim@yahoo.com (A.B.A.-N.); 2 Department of Pharmacy Practice, Faculty of Pharmacy, King Abdulaziz University, Jeddah 21589, Saudi Arabia; keljaaly@kau.edu.sa (K.E.); htbakhsh@kau.edu.sa (H.T.B.); 3 Department of Pharmaceutics, Faculty of Pharmacy, King Abdulaziz University, Jeddah 21589, Saudi Arabia; usamafahmy@hotmail.com

**Keywords:** colistin, nephrotoxicity, rats

## Abstract

The clinical value of colistin, a polymyxin antibiotic, is limited by its nephrotoxicity. Omeprazole is a commonly prescribed proton pump inhibitor. The current study aimed to evaluate the effects of the concomitant administration of omeprazole on colistin-induced nephrotoxicity in rats. Omeprazole significantly ameliorated colistin nephrotoxicity as evidenced by prevention in the rise in the serum level of creatinine, urea and cystactin C as well as urinary N-acetylglucosamine activity. This was confirmed by histological studies that indicated a decreased incidence of interstitial nephritis, degenerative cortical changes and collagen deposition. This was accompanied by the prevention of oxidative stress as omeprazole significantly inhibited the lipid peroxidation, glutathione depletion and enzymatic exhaustion of superoxide dismutase as well as catalase. Additionally, omeprazole inhibited the expression of interleukin-6 and tumor necrosis factor-α. Further, omeprazole inhibited the colistin-induced rise in Bax and the down-regulation of Bcl2 mRNA expression. An assessment of the serum levels of colistin revealed that omeprazole had no significant impact. However, it was observed that omeprazole significantly inhibited the accumulation of colistin in kidney tissues. In conclusion, omeprazole protects against colistin-induced nephrotoxicity. This can be attributed to, at least partly, omeprazole’s anti-oxidant, anti-inflammatory and anti-apoptotic activities in addition to its ability to prevent the toxic accumulation of colistin in kidneys.

## 1. Introduction

Colistin is a polymyxin antibiotic and a last-resort glycopeptide antibiotic that was approved for medical use several decades ago. It is available commercially as colistin sulfate for topical and oral use, and as colistimethate sodium (CMS, colistin sodium methanesulfonate) intended for parenteral and inhalation use [1]. CMS is considered a prodrug that is bioactivated in vivo to colistin [2,3]. However, colistin use was largely discontinued due to the high incidence of nephrotoxicity [1]. In a meta-analysis, the incidence of nephrotoxicity in patients who received colistin was reported to be around 36.2%. Other reports indicated that the incidence of colistin nephrotoxicity can be as high as 53.5% [4,5]. Yet, as multidrug-resistant bacterial infections have currently become more prevalent and are linked with increased mortality, colistin re-emerged as an emergency solution despite the raised concerns about its serious nephrotoxicity. Nephrotoxicity induced by colistin is dose-dependent and could develop into permanent kidney damage [6]. It is clinically manifested by increased serum levels of creatinine, as well as proteinuria, oliguria, or cylindruria [7].

Colistin nephrotoxicity is complex as it involves various cellular events, including oxidative, inflammatory, and apoptotic pathways [6,8]. It is characterized by the increased permeability of the tubular epithelial cell membrane, thereby resulting in water, anion, and cation influx causing cell swelling and lysis [6]. It was evidenced that these nephrotoxic effects are initiated upon the accumulation of colistin inside kidney cortical cells [9,10]. Hence, reducing the reabsorption of colistin by the proximal tubular cells was associated with a significant decrease in N-acetyl-β-D-glucosaminidase urinary excretion, an indicator of renal tubular dysfunction [9]. Additionally, a urinary metabolomic characterization revealed that rats given a single intraperitoneal injection of colistin had almost no signs of nephrotoxicity [11] Colistin was recently shown to be a substrate for the organic cation transporter 2 (OCTN2), which is a solute carrier membrane protein engaged in carnitine active cellular uptake. The stable transfection of HEK293 cells with the open reading frame of OCTN2 was shown to promote the accumulation of colistin over time compared to wild-type cells [12].

Omeprazole is a commonly prescribed proton pump inhibitor that decreases the amount of acid produced in the stomach. It is frequently used to treat symptoms of gastroesophageal hyperacidity, reflux esophagitis, and gastric and duodenal ulcers [13]. In a retrospective cohort study, the co-administration of proton pump inhibitors was reported to protect against cisplatin nephrotoxicity in cancer patients [14]. Interestingly, several studies indicated that omeprazole inhibits the OCTN2-mediated transport of cisplatin and metformin (a common substrate of OCTN2), providing evidence that omeprazole could ameliorate the nephrotoxicity instigated by other drugs by reducing their renal accumulation [15,16,17,18]. Furthermore, omeprazole was found to decrease oxidative stress and inflammation in various disease models [18,19,20]. Therefore, the present study aimed to investigate the effects of the concomitant administration of omeprazole on colistin-induced nephrotoxicity in rats.

## 2. Results

### 2.1. Assessment of Kidney Function

**Figure 1:**
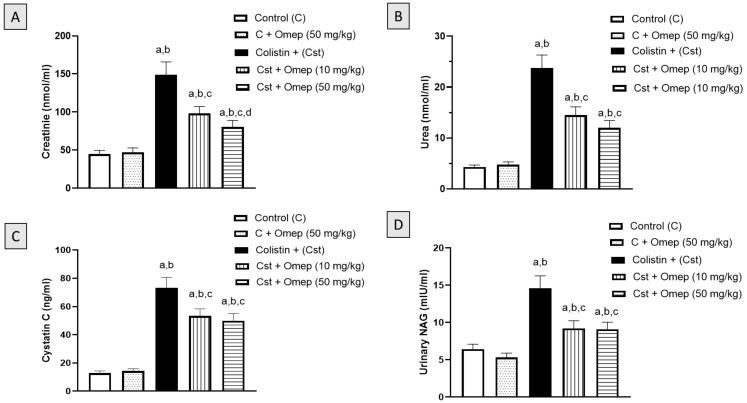
Effect of omeprazole on serum and urinary markers of nephrotoxicity induced by colistin in rats. (**A**): Serum creatinine level, (**B**): Serum urea level, (**C**): Serum cystatin C level, (**D**) Urinary NAG activity. Data are shown as M ± SD (*n* = 6). Omeprazole = Omep; Colistin = Cst. a: Significantly different from Control at *p* < 0.05; b: Significantly different from Omep (50 mg/kg) at *p* < 0.05; c: Significantly different from Cst at *p* < 0.05; d: Significantly different from Cst + Omep (10 mg/kg) at *p* < 0.05.

Figure 1A demonstrates that colistin injection resulted in a significant increase in serum creatinine level, of 332% as compared to the control group. Yet, this increase was significantly ameliorated by 34% and 46% when omeprazole was administered at 10 mg/kg and 50 mg/kg, respectively. Figure 1B,C show that treatment with colistin alone led to a 550% and 573% increase in serum urea and cystatin C levels compared to control values. However, the concurrent administration of omeprazole inhibited the rise in serum urea and cystatin C levels by 50% and 32% at 50 mg/kg, respectively. The activity of urinary NAG followed a comparable pattern as demonstrated in Figure 1D. The administration of omeprazole significantly prevented the rise in urinary NAG activity induced by colistin by 37% and 38% at both tested doses.

### 2.2. Histopathological Examination

**Figure 2:**
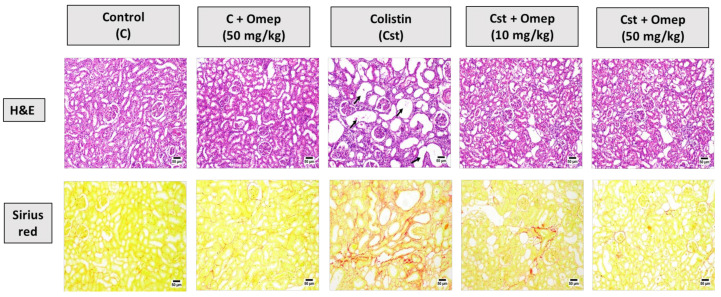
Effects of omeprazole on colistin-induced kidney injury as visualized using H&E (upper plates) and Sirius red staining (lower plates). Black arrows indicate diffuse interstitial nephritis, Red coloration in the Sirius red-stained sections indicates collagen deposition.

Microscopical examination of H&E-stained kidney sections (upper panel) from control group 1 revealed the absence of any detectable alterations in the renal cortex or medulla. Similarly, an apparently normal kidney architecture was detected in the omeprazole alone (50 mg/kg) group. Kidneys sections obtained from rats challenged with colistin showed marked histopathological alterations; both the renal cortex and medulla exhibited intense diffuse mononuclear inflammatory cells infiltration with mild fibrosis and collagen deposition in some instances. The renal tubules suffered degeneration and necrosis with the presence of numerous cystically dilated tubules. Some of the examined sections showed bluish areas of calcification. Some renal tubules contained an esinophilic cast. This was accompanied by fibrosis as indicated by collagen deposition as observed in picrosirius red-stained sections (lower panel). A moderate improvement was observed in the examined kidney sections from animals exposed to colistin and omeprazole (10 mg/kg). The renal cortex showed focal areas of mononuclear inflammatory cells aggregations. The renal tubules suffered from mild degenerative changes. Few tubules showed an esinophilic cast in the renal medulla. The best improvement was achieved in animals given colistin and omeprazole (50 mg/kg). The renal cortex was apparently normal in most of the examined sections with mild degeneration in some tubules and minor collagen deposition (Figure 2).

### 2.3. Assessment of Oxidative Status

**Figure 3:**
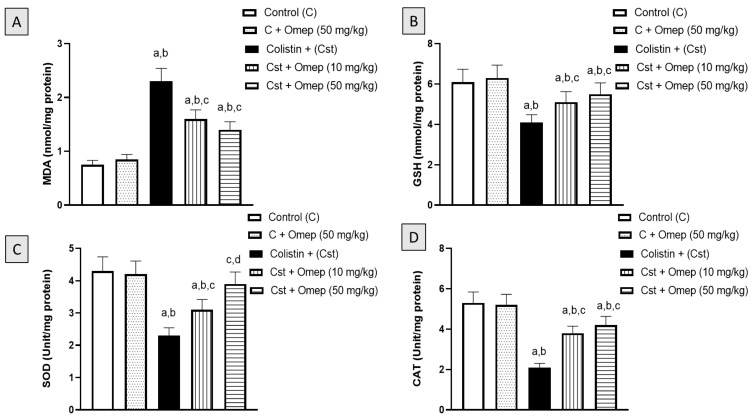
Effect of omeprazole on oxidative status in colistin-induced nephrotoxicity in rats. (**A**): MDA, (**B**): GSH, (**C**): SOD, (**D**) CAT. Data are shown as M ± SD (*n* = 6). Omeprazole = Omep; Colistin = Cst. a: Significantly different from Control at *p* < 0.05; b: Significantly different from Omep (50 mg/kg) at *p* < 0.05; c: Significantly different from Cst at *p* < 0.05; d: Significantly different from Cst + Omep (10 mg/kg) at *p* < 0.05.

The following experiments were carried out to examine the protective effects provided by omeprazole on oxidative status in colistin-induced nephrotoxicity in rats. Figure 3A indicates that MDA, a final product of lipid peroxidation, significantly increased due to colistin challenge by 207% of the control value. However, omeprazole ameliorated this rise associated with colistin by 30% and 40% at 10 mg/kg and 50 mg/kg, respectively. Regarding the GSH concentration, the groups that received omeprazole and colistin expressed significantly elevated GSH concentrations compared to the group that received colistin alone. GSH concentrations in the 10 mg/kg and 50 mg/kg omeprazole groups were 24% and 34% higher than the colistin-only group, respectively. Similarly, SOD and CAT activities in the groups that received omeprazole and colistin were significantly higher than the activities in the colistin-only group by 70% and 100% at a dose of 50 mg/kg, respectively.

### 2.4. Immunohistochemical Assessment of IL-6 and TNF-α Expression in Kidney Tissues

**Figure 4:**
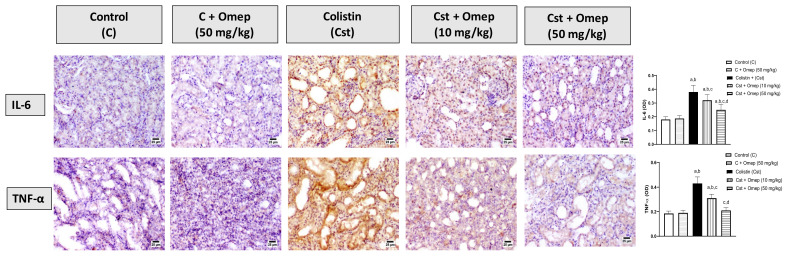
Effect of omeprazole on the expression of IL-6 and TNF-α. Immunohistochemical photomicrographs of kidney sections showing the effect of omeprazole on colistin-induced alterations in IL-6 and TNF-α. Data shown in bar charts are the Mean of optical densities ± SD (*n* = 6). Omeprazole = Omep; Colistin = Cst. a: Significantly different from Control at *p* < 0.05; b: Significantly different from Omep (50 mg/kg) at *p* < 0.05; c: Significantly different from Cst at *p* < 0.05; d: Significantly different from Cst + Omep (10 mg/kg) at *p* < 0.05.

The potential of omeprazole to ameliorate colistin-induced inflammation was assessed in stressed kidney tissues. The upper panel in Figure 4 shows that the co-administration of omeprazole significantly inhibited the elevated expression of IL-6 associated with colistin injection by 16% and 34% at 10 and 50 mg/kg, respectively. Moreover, the expression of TNF-α was significantly enhanced by colistin as can be seen in the bottom panel of Figure 4. Yet, omeprazole treatment significantly inhibited this rise by 28% and 51% at 10 and 50 mg/kg, respectively.

### 2.5. mRNA Expression of Bax and Bcl-2

**Figure 5:**
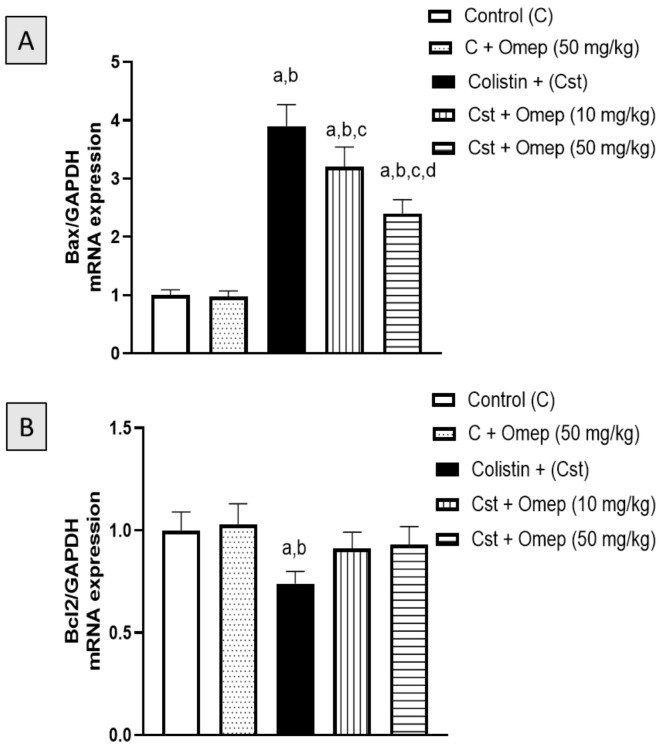
Effects of omeprazole treatment on colistin-induced alterations in Bax (**A**) and Bcl2 (**B**) mRNA expression in kidney tissues. Data shown in bar charts are the Mean of optical densities ± SD (*n* = 6). Omeprazole = Omep; Colistin = Cst. a, b, c or d: Statistically different from Control, colistin, colistin + omeprazole (10 mg/kg), or Cst + Omep (10 mg/kg), respectively at *p* < 0.05.

The anti-apoptotic potential of omeprazole was inspected by investigating Bax and Bcl2 mRNA expression in kidney tissues of animals challenged with colistin. As shown in Figure 5A, the increased mRNA expression of Bax induced by colistin was significantly inhibited by 18% and 39% with the co-administration of omeprazole at 10 mg/kg and 50 mg/kg, respectively. On the other hand, colistin challenge resulted in a significant decrease in the mRNA expression of Bcl2 by approximately 26% (Figure 5B). However, this decrease was significantly prevented by omeprazole treatment in a dose-unrelated manner.

### 2.6. Effect of Omeprazole on Colistin Serum and Kidney Concentration

**Figure 6:**
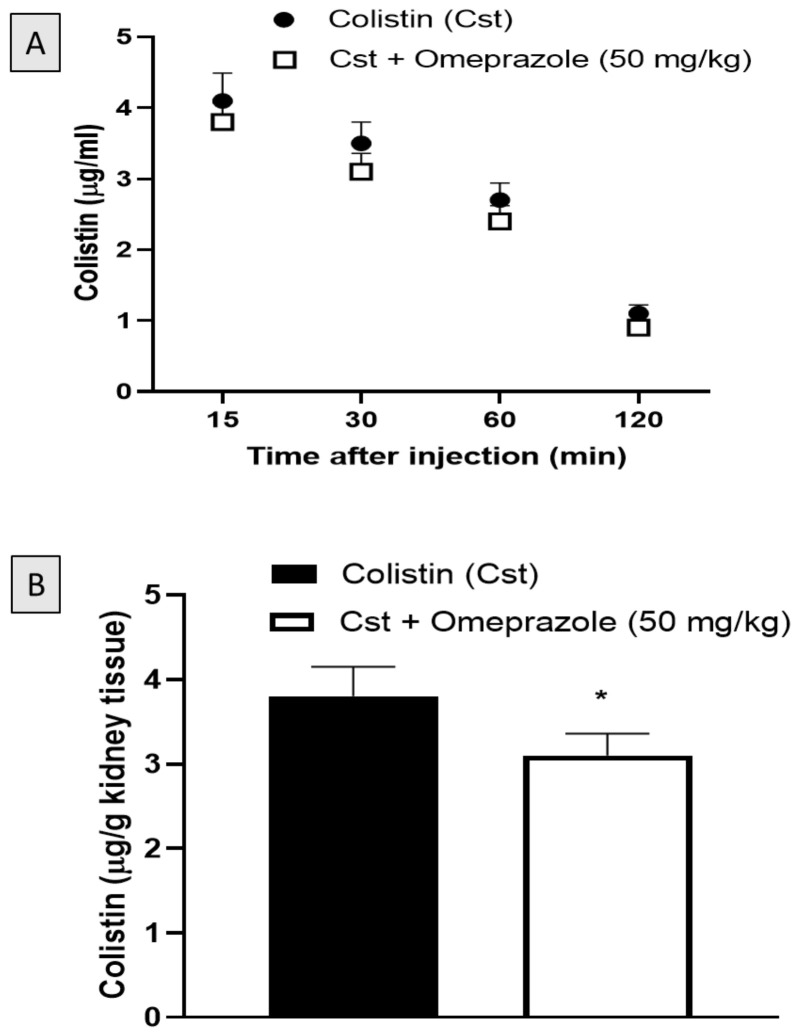
Plasma (**A**) and kidney concentration (**B**) of colistin after an IP injection either alone or with omeprazole (50 mg/kg). Data are shown as M ± SD (*n* = 6). * Significantly different from corresponding Cst value as determined by Student’s *t* test at *p* < 0.05.

As shown in Figure 6A, there were no significant differences in the plasma levels of colistin between rats that were injected with colistin alone or colistin combined with omeprazole treatment at all time intervals. Interestingly, the renal concentration of colistin in the omeprazole-treated rats was significantly reduced compared to the colistin alone group (Figure 6B).

## 3. Discussion

Adverse effects including serious nephrotoxicity are common with colistin treatment [1]. These deleterious side effects markedly limit the therapeutic value of colistin [4,5]. However, colistin is being increasingly utilized clinically due to the emergence and rapid spread of multidrug-resistant Gram-negative bacteria [6]. Hence, the identification of protective strategies to alleviate colistin-induced nephrotoxicity is essential. Colistin nephrotoxicity is a complex pathological process involving oxidative stress and inflammation and was reported to be initiated upon colistin renal accumulation [9,10]. Omeprazole, a proton pump inhibitor, has been shown to protect against nephrotoxicity in a multifaceted mechanism that involves the alleviation of oxidative stress, inflammation, and transporter-mediated drug accumulation in the kidney [15,16,18,20]. Hence, the current work was designed to evaluate the possible protective effects of omeprazole against colistin-induced nephrotoxicity in rats.

In this study, the biochemical and histopathological studies indicated that colistin nephrotoxicity was associated with a reduced glomerular filtration rate and collagen deposition. This is in line with the reported marker of colistin nephrotoxicity [21,22]. Interestingly, omeprazole alleviated such markers, thereby confirming its protective activity. Among the involved mechanisms in nephrotoxicity induced by colistin is the induction of oxidative stress resulting in severe renal injury [8]. The obtained findings in this study suggest a remarkable antioxidant potential of omeprazole, as indicated by the significant attenuation of pathological alterations induced by colistin to the markers of oxidative stress-tested, namely MDA, GSH, SOD, and CAT in kidney tissues. These findings are in agreement with several reports in the literature highlighting the significant antioxidant effects of omeprazole in addition to its acid-suppression action [23,24]. The growing body of evidence highlighting the pathological role of oxidative stress in colistin-induced renal injury suggests that the protective effects observed in this study involve the antioxidant properties of omeprazole [25].

Omeprazole treatment also protected against the colistin-induced increase in kidney inflammatory factors, namely, IL-6 and TNF-α. Inflammation plays a significant role in kidney injury induced by colistin [26]. These results are consistent with the reported anti-inflammatory activity of omeprazole against kidney injury induced by the nephrotoxic drug cisplatin [27]. Furthermore, the genetic deletion or pharmacological inhibition of pro-inflammatory mediators was demonstrated to reduce leukocyte infiltration, epithelial necrosis and apoptosis, and renal dysfunction associated with colistin administration [28,29]. Indeed, our results also demonstrated that colistin induced apoptotic changes in Bax and Bcl-2 mRNA expression. However, the concurrent administration of omeprazole significantly ameliorated these apoptotic changes associated with colistin exposure. In line with these results, omeprazole was shown to be protective against the apoptotic changes induced by cisplatin in the kidney, highlighting its potential as a nephroprotective agent [18].

Renal accumulation of colistin is a significant contributor to its nephrotoxicity [9,10]. Our data show that omeprazole significantly reduced the accumulated content of colistin in renal tissues, without apparent alterations in its serum levels. As omeprazole is a potent inhibitor of OCTN2, it can be deduced that the renal accumulation of mediated colistin was significantly inhibited by the concurrent administration of omeprazole [11,12,16,17]. These findings are also in agreement with a previous report demonstrating the potential of omeprazole to inhibit the OCTN2-mediated renal accumulation of cisplatin in rats [18]. The possibility of reduced colistin efficacy by the co-administration of omeprazole cannot be excluded. In addition, cases of urinary tract inflammation such as pyelonephritis may also lead to the reduced ability of omeprazole to mitigate colistin-induced kidney injury. Remarkably, reducing renal accumulation of colistin was consistently found to alleviate its nephrotoxicity [9,30].

## 4. Materials and Methods

### 4.1. Chemicals

Colistin, polymyxin B sulfate, and omeprazole (Omep) were purchased from Sigma-Aldrich (St. Louis, MO, USA). All other chemicals were of the highest purity.

### 4.2. Animals

Male Wistar rats (210–240 g) were obtained from the vivarium of Faculty of Pharmacy. Animals were kept in an air-conditioned facility (a temperature of 22 ± 2 °C and humidity of 60–70%) under a dark/light cycle of 12 h:12 h with free access to tap water and standard food pellets. Animal handling procedures and protocol were permitted by the Research Ethics Committee (REC), Faculty of Pharmacy, KAU, Saudi Arabia (Reference # PH-1443-10).

### 4.3. Experimental Protocol

Thirty rats were randomly separated into 5 groups (*n* = 6) as follows: Group 1 (Control group, C) received daily oral and IP normal saline (dosing volume 5 mL/kg); Group 2 (Omeprazole 50 mg/kg) received oral omeprazole (50 mg/kg/day) and IP injection of normal saline; Group 3 (Colistin) received daily oral normal saline and IP colistin at a dose of 1000,000 IU/kg/day; Group 4 (Colistin + Omeprazole 10 mg/kg) received daily oral omeprazole (10 mg/kg/day) and IP colistin at a dose of 1000,000 IU/kg/day; Group 5 (Colistin + Omeprazole 50 mg/kg) received daily oral omeprazole (50 mg/kg/day) and IP colistin at a dose of 1000,000 IU/kg/day. All treatments were continued for 10 consecutive days. Oral omeprazole doses were administered 30 min before IP injections of colistin. The doses and regimen used were selected based on a pilot experiment and were consistent with those in the published literature [31,32,33]. On day 10, each rat was placed in a metabolic cage for 24h-urine collection. On day 11, urine was collected and rats were anesthetized using IP ketamine (100 mg/kg) and xylazine (10 mg/kg). Blood samples were obtained from the retroorbital plexus and centrifuged at 3000 rpm for 10 min at 4 °C to obtain sera that were stored at −80 °C for analysis. This was followed by decapitation, abdomens were opened and kidneys were rapidly dissected and rinsed gently with cold saline. Sections of the kidney were either fixed in 10% formalin or other parts were frozen in liquid nitrogen and kept at −80 °C directly or kept in an RNase inactivating and stabilizing medium for 24 h followed by storage at −80 °C for subsequent biochemical and PCR analyses.

For the assessment of blood and kidney colistin concentration, 12 additional male rats were divided into two groups. Animals in the first group were given saline orally 30 min before colistin (300,000 IU/kg, IP), while animals in the second group were given omeprazole (50 mg/kg, orally) 30 min before colistin (300,000 IU/kg, IP). Under ketamine/xylazine anaesthesia, blood samples were collected from the retro-orbital plexus at 15, 30, 60 and 120 min after colistin administration. Then, animals were euthanized via decapitation and kidneys were rapidly dissected and kept at −80 °C for colistin determination.

### 4.4. Biochemical Assays of Renal Function

Commercial kits were used to assess the serum level of creatinine (Cat. No. E4370-100, BioVision, Milpitas, CA, USA), urea (Cat. No. ab83362, Abcam, Cambridge, UK) and cystatin C (Cat. No. CSB-E08385r, CUSABIO, Fannin, TX, USA). Additionally, the urinary activity of N-acetyl-β-D-glucosaminidase (NAG) was assessed using a commercial kit (Cat. No. CSB-E07443r, CUSABIO, Fannin, TX, USA). The BCA Protein Assay Kit (Cat. No. D49328, Sigma-Aldrich, St. Louis, MO, USA) was used to assess the protein concentration in kidney homogenates.

### 4.5. Histopathological Examination

Kidneys were kept in 10% neutral buffered formalin for 24 h. This was followed by paraffinization. Sections of 5 µm were either stained with Hematoxylin and Eosin (H&E) or Sirius red and then visualized using a light microscope (OPTIKA, B-350, Ponteranica, Bergamo, Italy). Additional unstained tissue sections collected on positively charged slides were kept for subsequent immunohistochemical testing.

### 4.6. Assessment of Oxidative Stress Markers

The homogenization of kidney tissues was performed in X10 volumes of phosphate-buffered saline (PBS 100 mM, pH 7.4). After centrifugation for 20 min at 10,000× *g* at a temperature of 4 °C, supernatants were utilized for the assessment of oxidative stress markers using colorimetric kits for reduced glutathione (GSH), malondialdehyde (MDA), and enzymatic activity of superoxide dismutase (SOD) and catalase (CAT) according to the manufacturer instructions (Cat. No. GR2511, MD2529, SD2521 and CA2517, Biodiagnostic, Giza, Egypt).

### 4.7. Immunohistochemistry Evaluation of Inflammatory Markers

Sections of renal tissues were deparaffinized and then rehydrated using serial dilutions of ethanol before boiling for 12 min in citrate buffer (0.1 M, pH 6.0) and incubating for 120 min in tris-buffered saline with 5% bovine serum albumin (BSA). Tissue sections were then incubated for 12 h at 4 °C with the primary antibodies IL-6 and TNFα (Cat. No. ab9324 and ab220210, respectively, Abcam, Cambridge, UK). After tissue washing using TBS, the Anti-Mouse HRP-DAB Cell and Tissue Staining kit was utilized for primary antibody detection (Cat. No. CTS002 R&D systems, Minneapolis, MN, USA). Quantification was carried out using ImageJ 1.52a (NIH, Bethesda, MD, USA).

### 4.8. Quantitative Polymerase Chain Reaction (qPCR) for Bax and Bcl-2

RNA was isolated from the kidney tissues using TRIzol and then RNA purity was confirmed based on the A260/A280 ratio. For first-strand cDNA synthesis and the quantification of mRNA, Omniscript RT kit and SYBR Green Master Mix (Cat. No. 205113 and 180830, Qiagen, MD, USA, respectively) were utilized according to their provided instructions. For Bax, the following forward and reverse primers were used: 5′CCTGAGCTGACCTTGGAGCA and 5′GGTGGTTGCCCTTTTCTACT, respectively. For Bcl2 and β-actin forward, the primers were 5′TGATAACCGGGAGATCGTGA and 5′TCCGTCGCCGGTCCACACCC, respectively, while the reverse primers were 5′AAAGCACATCCAATAAAAAGC and 5′TCACCAACTGGGACGATATG, respectively [34]. β-Actin was used for normalization, and data analysis was carried out according to the ΔΔCT method [35].

### 4.9. Assessment of Serum and Kidney Colistin Using Liquid Chromatography-Mass Spectrometry

An Agilent 1200 system with a column compartment (Zorbax Eclipse Plus C18 column, 3.5 mm, 4.6 × 100 mm), quaternary pump and autosampler was used (Agilent Technologies, Waldbronn, Germany). The system was equipped with an Agilent 6420 triple quadrupole mass spectrometer operated by the software MassHunter ver B.03.01. The triple quadrupole mass spectrometer conditions were optimized. Acetonitrile: 0.1% formic in water (30: 70, *v*/*v*) with a flow rate of 0.4 mL/min was used to elute the colistin sulphate. Polymyxin B sulphate was used as an internal standard. Samples were pre-treated as previously prescribed [36].

### 4.10. Statistical Analysis

Data are represented as mean ± SD. Unless otherwise indicated, data were analyzed using one-way ANOVA followed by Tukey as a post hoc test using GraphPad Prism ver 8.1 (GraphPad Software, La Jolla, CA, USA). A *p* < 0.05 was taken as the cut-off value for significance.

## 5. Conclusions

Conclusively, the nephroprotective effects of omeprazole observed in this study could be at least partially attributable to its antioxidant, anti-inflammatory and anti-apoptotic properties and its aptitude to diminish the accumulation of colistin in kidney tissue.

## Data Availability

Data is contained within the article.

## References

[1] Li J., Nation R.L., Turnidge J.D., Milne R.W., Coulthard K., Rayner C.R., Paterson D.L. (2006). Colistin: The Re-Emerging Antibiotic for Multidrug-Resistant Gram-Negative Bacterial Infections. Lancet. Infect. Dis..

[2] Bergen P.J., Li J., Rayner C.R., Nation R.L. (2006). Colistin Methanesulfonate Is an Inactive Prodrug of Colistin against Pseudomonas Aeruginosa. Antimicrob. Agents Chemother..

[3] Mendes C.A.C., Burdmann E.A. (2009). Polymyxins—Review with Emphasis on Nephrotoxicity. Rev. Assoc. Med. Bras..

[4] Eljaaly K., Bidell M.R., Gandhi R.G., Alshehri S., Enani M.A., Al-Jedai A., Lee T.C. (2021). Colistin Nephrotoxicity: Meta-Analysis of Randomized Controlled Trials. Open Forum Infect. Dis..

[5] Spapen H., Jacobs R., van Gorp V., Troubleyn J., Honoré P.M. (2011). Renal and Neurological Side Effects of Colistin in Critically Ill Patients. Ann. Intensive Care.

[6] Ordooei Javan A., Shokouhi S., Sahraei Z. (2015). A Review on Colistin Nephrotoxicity. Eur. J. Clin. Pharmacol..

[7] Hartzell J.D., Neff R., Ake J., Howard R., Olson S., Paolino K., Vishnepolsky M., Weintrob A., Wortmann G. (2009). Nephrotoxicity Associated with Intravenous Colistin (Colistimethate Sodium) Treatment at a Tertiary Care Medical Center. Clin. Infect. Dis..

[8] Dai C., Li J., Tang S., Li J., Xiao X. (2014). Colistin-Induced Nephrotoxicity in Mice Involves the Mitochondrial, Death Receptor, and Endoplasmic Reticulum Pathways. Antimicrob. Agents Chemother..

[9] Suzuki T., Yamaguchi H., Ogura J., Kobayashi M., Yamada T., Iseki K. (2013). Megalin Contributes to Kidney Accumulation and Nephrotoxicity of Colistin. Antimicrob. Agents Chemother..

[10] Yun B., Azad M.A.K., Wang J., Nation R.L., Thompson P.E., Roberts K.D., Velkov T., Li J. (2015). Imaging the Distribution of Polymyxins in the Kidney. J. Antimicrob. Chemother..

[11] Jeong E.S., Kim G., Moon K.S., Kim Y.B., Oh J.H., Kim H.S., Jeong J., Shin J.G., Kim D.H. (2016). Characterization of Urinary Metabolites as Biomarkers of Colistin-Induced Nephrotoxicity in Rats by a Liquid Chromatography/Mass Spectrometry-Based Metabolomics Approach. Toxicol. Lett..

[12] Visentin M., Gai Z., Torozi A., Hiller C., Kullak-Ublick G.A. (2017). Colistin Is Substrate of the Carnitine/Organic Cation Transporter 2 (OCTN2, SLC22A5). Drug Metab. Dispos..

[13] Targownik L.E., Metge C., Roos L., Leung S. (2007). The Prevalence of and the Clinical and Demographic Characteristics Associated with High-Intensity Proton Pump Inhibitor Use. Am. J. Gastroenterol..

[14] Ikemura K., Oshima K., Enokiya T., Okamoto A., Oda H., Mizuno T., Ishinaga H., Muraki Y., Iwamoto T., Takeuchi K. (2017). Co-Administration of Proton Pump Inhibitors Ameliorates Nephrotoxicity in Patients Receiving Chemotherapy with Cisplatin and Fluorouracil: A Retrospective Cohort Study. Cancer Chemother. Pharmacol..

[15] Hiramatsu S.I., Ikemura K., Fujisawa Y., Iwamoto T., Okuda M. (2020). Concomitant Lansoprazole Ameliorates Cisplatin-Induced Nephrotoxicity by Inhibiting Renal Organic Cation Transporter 2 in Rats. Biopharm. Drug Dispos..

[16] Hacker K., Maas R., Kornhuber J., Fromm M.F., Zolk O. (2015). Substrate-Dependent Inhibition of the Human Organic Cation Transporter OCT2: A Comparison of Metformin with Experimental Substrates. PLoS ONE.

[17] Nies A.T., Hofmann U., Resch C., Schaeffeler E., Rius M., Schwab M. (2011). Proton Pump Inhibitors Inhibit Metformin Uptake by Organic Cation Transporters (OCTs). PLoS ONE.

[18] Gao H., Zhang S., Hu T., Qu X., Zhai J., Zhang Y., Tao L., Yin J., Song Y. (2019). Omeprazole Protects against Cisplatin-Induced Nephrotoxicity by Alleviating Oxidative Stress, Inflammation, and Transporter-Mediated Cisplatin Accumulation in Rats and HK-2 Cells. Chem. Biol. Interact..

[19] Patil A.S., Singh A.D., Mahajan U.B., Patil C.R., Ojha S., Goyal S.N. (2019). Protective Effect of Omeprazole and Lansoprazole on β-Receptor Stimulated Myocardial Infarction in Wistar Rats. Mol. Cell. Biochem..

[20] Özay R., Türkoğlu M.E., Gürer B., Dolgun H., Evirgen O., Ergüder B.İ., Hayırlı N., Gürses L., Şekerci Z. (2017). The Protective Effect of Omeprazole Against Traumatic Brain Injury: An Experimental Study. World Neurosurg..

[21] Lee Y.J., Wi Y.M., Kwon Y.J., Kim S.R., Chang S.H., Cho S. (2015). Association between Colistin Dose and Development of Nephrotoxicity. Crit. Care Med..

[22] Mirjalili M., Mirzaei E., Vazin A. (2022). Pharmacological Agents for the Prevention of Colistin-Induced Nephrotoxicity. Eur. J. Med. Res..

[23] Lapenna D., de Gioia S., Ciofani G., Festi D., Cuccurullo F. (1996). Antioxidant Properties of Omeprazole. FEBS Lett..

[24] Abed M.N., Alassaf F.A., Jasim M.H.M., Alfahad M., Qazzaz M.E. (2020). Comparison of Antioxidant Effects of the Proton Pump-Inhibiting Drugs Omeprazole, Esomeprazole, Lansoprazole, Pantoprazole, and Rabeprazole. Pharmacology.

[25] Gai Z., Samodelov S.L., Kullak-Ublick G.A., Visentin M. (2019). Molecular Mechanisms of Colistin-Induced Nephrotoxicity. Molecules.

[26] Miyasato Y., Yoshizawa T., Sato Y., Nakagawa T., Miyasato Y., Kakizoe Y., Kuwabara T., Adachi M., Ianni A., Braun T. (2018). Sirtuin 7 Deficiency Ameliorates Cisplatin-Induced Acute Kidney Injury Through Regulation of the Inflammatory Response. Sci. Rep..

[27] Gao H., Wang X., Qu X., Zhai J., Tao L., Zhang Y., Song Y., Zhang W. (2020). Omeprazole Attenuates Cisplatin-Induced Kidney Injury through Suppression of the TLR4/NF-ΚB/NLRP3 Signaling Pathway. Toxicology.

[28] Ramesh G., Reeves W.B. (2003). TNFR2-Mediated Apoptosis and Necrosis in Cisplatin-Induced Acute Renal Failure. Am. J. Physiol. Renal Physiol..

[29] Sahu B.D., Kumar J.M., Sistla R. (2015). Baicalein, a Bioflavonoid, Prevents Cisplatin-Induced Acute Kidney Injury by Up-Regulating Antioxidant Defenses and Down-Regulating the MAPKs and NF-ΚB Pathways. PLoS ONE.

[30] Yousef J.M., Chen G., Hill P.A., Nation R.L., Li J. (2011). Melatonin Attenuates Colistin-Induced Nephrotoxicity in Rats. Antimicrob. Agents Chemother..

[31] Kobayashi T., Ohta Y., Inui K., Yoshino J., Nakazawa S. (2002). Protective Effect of Omeprazole against Acute Gastric Mucosal Lesions Induced by Compound 48/80, a Mast Cell Degranulator, in Rats. Pharmacol. Res..

[32] Ceylan B., Ozansoy M., Kılıç Ü., Yozgat Y., Ercan Ç., Yıldız P., Aslan T. (2018). N-Acetylcysteine Suppresses Colistimethate Sodium-Induced Nephrotoxicity via Activation of SOD2, ENOS, and MMP3 Protein Expressions. Ren. Fail..

[33] Lee D.Y., Lee M.G., Shin H.S., Lee I. (2007). Changes in Omeprazole Pharmacokinetics in Rats with Diabetes Induced by Alloxan or Streptozotocin: Faster Clearance of Omeprazole Due to Induction of Hepatic CYP1A2 and 3A1. J. Pharm. Pharm. Sci..

[34] Sirwi A., Shaik R.A., Alamoudi A.J., Eid B.G., Kammoun A.K., Ibrahim S.R.M., Mohamed G.A., Abdallah H.M., Abdel-Naim A.B. (2021). Mokko Lactone Attenuates Doxorubicin-Induced Hepatotoxicity in Rats: Emphasis on Sirt-1/FOXO1/NF-ΚB Axis. Nutrients.

[35] Livak K.J., Schmittgen T.D. (2001). Analysis of Relative Gene Expression Data Using Real-Time Quantitative PCR and the 2(-Delta Delta C(T)) Method. Methods.

[36] Matar K.M., Al-Refai B. (2020). Quantification of Colistin in Plasma by Liquid Chromatography-Tandem Mass Spectrometry: Application to a Pharmacokinetic Study. Sci. Rep..

