# Correction: Nasrullah et al. Omeprazole Prevents Colistin-Induced Nephrotoxicity in Rats: Emphasis on Oxidative Stress, Inflammation, Apoptosis and Colistin Accumulation in Kidneys. *Pharmaceuticals* 2022, *15*, 782

**DOI:** 10.3390/ph17040540

**Published:** 2024-04-22

**Authors:** Mohammed Z. Nasrullah, Khalid Eljaaly, Thikryat Neamatallah, Usama A. Fahmy, Abdulmohsin J. Alamoudi, Hussain T. Bakhsh, Ashraf B. Abdel-Naim

**Affiliations:** 1 Department of Pharmacology and Toxicology, Faculty of Pharmacy, King Abdulaziz University, Jeddah 21589, Saudi Arabia; taneamatallah@kau.edu.sa (T.N.); ajmalamoudi@kau.edu.sa (A.J.A.); abnaim@yahoo.com (A.B.A.-N.); 2 Department of Pharmacy Practice, Faculty of Pharmacy, King Abdulaziz University, Jeddah 21589, Saudi Arabia; keljaaly@kau.edu.sa (K.E.); htbakhsh@kau.edu.sa (H.T.B.); 3 Department of Pharmaceutics, Faculty of Pharmacy, King Abdulaziz University, Jeddah 21589, Saudi Arabia; usamafahmy@hotmail.com

**Error in Figure**

In the original publication [1], there was a mistake in Figure 2. There was an error in the sub-image of the first row, last column. The corrected Figure 2 appears below. The authors state that the scientific conclusions are unaffected. This correction was approved by the Academic Editor. The original publication has also been updated.


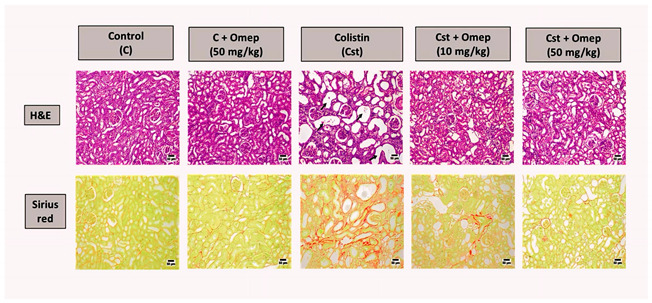


## References

[1] Nasrullah M.Z., Eljaaly K., Neamatallah T., Fahmy U.A., Alamoudi A.J., Bakhsh H.T., Abdel-Naim A.B. (2022). Omeprazole Prevents Colistin-Induced Nephrotoxicity in Rats: Emphasis on Oxidative Stress, Inflammation, Apoptosis and Colistin Accumulation in Kidneys. Pharmaceuticals.

